# IoT and Blockchain for Support for Smart Contracts Through TpM

**DOI:** 10.3390/s25165001

**Published:** 2025-08-13

**Authors:** Renan Yamaguti, Luiz Carlos B. C. Ferreira, Lucas Lui Motta, Raphael Montali Assumpção, Omar C. Branquinho, Gustavo Iervolino, Paulo Cardieri

**Affiliations:** 1 Department of Communications, School of Electrical and Computer Engineering, University of Campinas (FEEC), Campinas 13083-852, SP, Brazil; l192701@dac.unicamp.br (L.L.M.); r075126@dac.unicamp.br (R.M.A.); omarc@unicamp.br (O.C.B.); cardieri@unicamp.br (P.C.); 2 Federal Institute of Education, Science, and Technology of Southern Minas Gerais, Poços de Caldas Campus, Poços de Caldas 37713-100, MG, Brazil; l144401@dac.unicamp.br; 3 Department of Hardware Design, Instituto de Pesquisa Eldorado, Campinas 13083-898, SP, Brazil; gustavo.morais@eldorado.org.br

**Keywords:** Internet of things, bslockchain, smart contracts

## Abstract

This paper investigates the integration of Internet of things (IoT) technology with blockchain to enhance transparency, accountability, and operational efficiency in smart contract execution for IoT ecosystems. The proposed approach extends the Three-Phase Methodology (TpM) by introducing an innovative entity, the IoT Operator, which acts as a custody caretaker, contract enforcer, and mediator. By leveraging blockchain’s secure and immutable ledger, the IoT Operator ensures the reliable monitoring and governance of IoT applications. A PoC implementation conducted at the Eldorado Research Institute demonstrates the methodology’s effectiveness, realizing a significant reduction of 95.83% in equipment search time. This work highlights the practical advantages of integrating blockchain and IoT within a structured framework, emphasizing the need for tailored, application-specific solutions rather than generic decentralization. The findings offer actionable guidelines for implementing blockchain in IoT systems, paving the way for more secure, efficient, and resilient IoT applications.

## 1. Introduction

The Internet of things (IoT) is revolutionizing diverse industries, fostering an ever-expanding market fueled by substantial investments. Reports predict a further surge in the upcoming years, driven by the ubiquitous application of IoT across various knowledge domains [1].

Current methodologies for crafting IoT solutions, exemplified by TpM [2], prioritize the enterprise perspective over mere processes. This emphasis stems from the inherent entrepreneurial nature of IoT needs. The technologies employed become mere tools in achieving the desired solution, a vital distinction that sets TpM apart.

Enhanced productivity, a cornerstone of IoT solutions, hinges on meticulous monitoring and control. This necessitates a two-pronged approach, encompassing both process and enterprise aspects. Consider a factory: The production sector and the broader corporation represent distinct entities within the system. Their interactions are governed by contracts and commitments, which are crucial for achieving tangible or intangible gains [3].

The emergence of smart contracts, powered by blockchain technology, offers a compelling solution for managing these contracts and commitments within the IoT landscape. This technology, already explored in conjunction with IoT by companies like Walmart, presents a secure and transparent platform for governing relationships [4].

Arbitration becomes a crucial element in this scenario. A trusted, external entity, dubbed the IoT Operator, is proposed to fulfill this role. This entity, independent of the parties involved in the IoT solution, would possess the impartiality and experience to effectively resolve conflicts. Additionally, the IoT Operator would equip these parties with tools for accessing critical information in an objective, secure, and transparent manner, facilitating informed decision-making.

Blockchain’s inherent characteristics, such as auditability, transparency, immutability, and non-repudiation, make it ideal for developing secure and reliable IoT applications. Smart contracts further enhance this value proposition by streamlining business rule execution and work relationships, eliminating ambiguities and misinterpretations.

While various approaches have been explored for integrating blockchain and smart contracts into IoT, a standardized methodology has yet to be established [5]. This work presents an evolution of TpM [6], embedding blockchain and smart contracts from the initial stages of IoT solution development to ensure transparency, accountability, and operational efficiency. By introducing the IoT Operator, this enhanced TpM framework provides a structured approach for secure, auditable, and automated IoT deployments, making it adaptable for projects of varying sizes and complexities.

To validate the proposed methodology, the PoC was conducted at the Eldorado Research Institute. The goal was to improve equipment tracking and custody management by replacing manual, paper-based tracking with a blockchain-backed system. The results demonstrated significant gains in operational efficiency. The time required to locate misplaced equipment was reduced from an average of 2 h to just 5 min, achieving a 95.83% improvement. This directly minimized downtime, allowing personnel to focus on productive tasks instead of searching for missing assets. Additionally, the IoT Operator ensured accountability by linking every asset interaction to an authenticated user, reducing unauthorized equipment usage and potential losses. The auditability of transactions enhanced trust between stakeholders, simplifying compliance with internal policies and external regulations. The automated contract execution prevented delays and inconsistencies in equipment handovers, further streamlining operations. These improvements highlight the potential of blockchain-enabled IoT governance to transform asset management, security, and operational workflows in large-scale deployments.

## 2. Related Work

### 2.1. TpM

The IoT is a melting pot of diverse fields, drawing in professionals from engineering, data science, security, and beyond. This rich tapestry of expertise promises immense potential, but navigating the unique skills and perspectives can be challenging. Integrating these varied professionals seamlessly to ensure effective collaboration becomes a crucial challenge, demanding innovative strategies to bridge the divide and unlock the full symphony of the IoT’s collaborative power.

To address this issue, ref. [2] proposed a new methodology for project development in IoT, called the Three-Phase Methodology for IoT Project Development (TpM-Pro). TpM-Pro divides IoT development into three major recursive phases:

- Phase 1: Considering the business;
- Phase 2: Gathering the requirements;
- Phase 3: The implementation.

The TpM-Pro methodology for IoT projects prioritizes a deep understanding of the business at the outset. Its focus is on ensuring that the solution delivers maximum value, exceeding customer expectations. This contrasts with other methodologies that may prioritize technical aspects first.

Phase 1 of TpM-Pro delves into the following:

- Business: Each project’s specific needs and goals are identified, even within similar industries, avoiding generic solutions.
- Things: The objects or entities to be monitored, measured, or controlled are clearly defined, whether physical or virtual.
- Specialist: Experts are identified to provide insights and support decision-making.
- Business rules: The rules influencing business operations are analyzed and incorporated into the solution development, guaranteeing alignment with customer needs.

Ref. [7] proposed the TpM-IoT-Canvas, a visual tool inspired by Project Model Canvas (PM-Canvas) to collaboratively plan IoT projects. It uses sticky notes on a board to simplify the process of understanding the business and solution through eight key blocks: business, justification, benefits, product, things, solution requirements, client, and team. These blocks are connected, helping identify inconsistencies and track the impact of changes on different aspects of the project. Phase 1 culminates in a “Business Report” summarizing the findings and feeding into Phase 2. The approval of this report determines if the project moves forward.

Phase 2 of TpM-Pro delves into detailed requirements once the business objectives are understood. The requirements are broken down into two major categories: Functional (tasks and services) and Non-Functional (technical standards and system updates).

The process follows the IoT-OSRM reference model [8] in a top-down approach, from business-level needs to hardware specifications. Information is documented for each level:

- Level 6 (display): How information is presented (tables, graphs, and alerts).
- Level 5 (abstraction): Converting raw data into meaningful insights using expert knowledge and techniques like AI.
- Level 4 (storage): Where and how data are stored (cloud and client system), considering factors like security and redundancy.
- Level 3 (border): The element connecting the solution to the internet, requiring careful design and consideration of advanced strategies like software-defined networks.
- Level 2 (connectivity): Wired, wireless, or hybrid connection between “things” and the border element, chosen based on factors like cost, environment, and reliability.
- Level 1 (sensor/actuator node): Capabilities expected from sensors and actuators based on client needs, potentially involving local processing and edge/fog computing.

Security and privacy are addressed throughout the process, with specific requirements defined at each level. No single tool guarantees this, but a combination of techniques ensures overall protection. The “Requirements Report” summarizes all findings and is validated before moving to Phase 3. If not approved, Phase 2 iterates to resolve issues.

Phase 3 of TpM+ follows the same reference model as Phase 2 [8] but is implemented using a bottom-up approach. It starts at Level 1 (sensor/actuator node), selecting and integrating hardware components, followed by Level 2 (connectivity), where communication protocols and transceivers are defined. At Level 3 (border), edge computing infrastructure is established, linking local and cloud environments. Moving up, Level 4 (storage) determines the data management strategy, while Level 5 (abstraction) applies data processing techniques such as machine learning to generate insights. Finally, Level 6 (display) presents processed information to users through various interfaces. This structured implementation ensures seamless integration and scalability while maintaining consistency with the Phase 2 reference model.

Security and privacy measures are implemented throughout, from physical sensor/actuator security to user access controls on the display. TpM-Pro allows teams to leverage existing documentation and implementation techniques while maintaining a structured approach that prioritizes business needs. Phase 3 transforms requirements into a working solution, emphasizing flexibility and adaptability while ensuring security, privacy, and business alignment.

Despite its effectiveness in guiding IoT development, TpM-Pro lacks mechanisms that handle the following:

- Decentralized security and data integrity—traditional IoT deployments rely on centralized cloud architectures, creating vulnerabilities such as data breaches, single points of failure, and limited auditability.
- Automated governance and compliance—the methodology does not incorporate tools to enforce business rules, legal regulations, or service-level agreements (SLAs) in an automated and verifiable manner.
- Identity and access management—IoT devices are vulnerable to identity spoofing, unauthorized access, and other security threats, requiring a decentralized authentication mechanism.

Given these limitations, blockchain and smart contracts have emerged as essential tools for addressing security, trust, and automation challenges in IoT development. However, integrating these technologies into a structured IoT framework requires a dedicated entity capable of orchestrating decentralized security, automating governance, and ensuring seamless interoperability across diverse systems. This is where the IoT Operator becomes indispensable. By acting as a trusted intermediary, the IoT Operator mitigates the risks associated with centralized architectures, enforces compliance through smart contracts, and establishes a transparent, verifiable ecosystem for IoT deployments. Through its roles as a custody caretaker, contract enforcer, and mediator, the IoT Operator provides the necessary infrastructure to ensure secure, accountable, and automated IoT operations within the TpM+ framework.

### 2.2. Blockchain

While the concept of blockchain may seem novel, its roots lie in the work of [9]. In 1991, they proposed a groundbreaking method for timestamping documents in a tamper-proof manner using a cryptographically secure chain of blocks [9]. This system relied on cryptographic hash functions and Merkle trees [10] to create a secure record of certified documents within each block. While this technology predates Bitcoin, it gained widespread attention only after its innovative application in the cryptocurrency introduced by [11].

The widely used term “blockchain” was coined by [11], combining the initial concepts of “block” and “chain”. A block encapsulates information such as transactions, while the chain represents the tamper-proof links between these blocks secured by cryptographic hashes.

Beyond its initial application in cryptocurrencies, blockchain’s transformative potential lies in its ability to securely store and verify digital assets across various domains. By utilizing a distributed, cryptographically linked chain of blocks, blockchain offers tamper-proof audit trails, enhanced transparency, and increased trust in data provenance. This technology holds significant implications for revolutionizing various sectors, including finance, supply chain management, healthcare, and governance [12].

Blockchain operates on a decentralized consensus mechanism, eliminating the reliance on a centralized entity for transaction validation. Instead, designated nodes, acting as miners, utilize cryptographic algorithms and established consensus protocols to collectively verify the legitimacy of transactions. Validated transactions are then grouped into blocks and cryptographically linked to form an immutable chain of records. The sequential nature of the chain ensures that the execution of any transaction depends on the successful execution of all preceding transactions, establishing a robust system of trust and data integrity [13].

Driven by advancements in blockchain technology, major players like IBM [4] are actively developing more powerful, reliable, and cost-effective platforms, paving the way for wider industrial adoption. For instance, blockchain adoption has significantly reduced object tracking time in logistics and supply chain applications, demonstrating its efficiency and scalability.

The convergence of the IoT and blockchain technology is poised to revolutionize various industries. Blockchain’s secure and immutable ledger system offers unique advantages in addressing security concerns, empowering user control, and improving data management within the complex landscape of interconnected devices.

Traditional concerns surrounding data security in resource-constrained IoT devices are mitigated by blockchain’s tamper-proof storage and auditability, as highlighted by [14]. This fosters trust and transparency in applications such as secure communication between devices and centralized platforms, which are crucial for efficient and reliable data-driven operations [15]. In addition, users are empowered with greater control over their data through blockchain’s permission management capabilities. This enhances data ownership and privacy by allowing users to decide who can access their data and for what purposes, such as controlling access to personal health information collected by wearables [16].

The synergy between IoT and blockchain is already driving real-world advancements. In supply chain management, blockchain-powered IoT solutions enhance transparency by tracking the movement of goods and ensuring product origins, quality, and ethical sourcing throughout the entire supply chain, as demonstrated by various pilot projects [16]. Similarly, integrating blockchain with smart grids facilitates secure and transparent energy trading, enabling peer-to-peer transactions and efficient resource management [17]. Furthermore, blockchain offers secure storage and access control for sensitive medical data in the healthcare sector, empowering patients with ownership and improving data management practices [15].

Several studies have explored the integration of blockchain technology with IoT applications to enhance security, transparency, and trust. For instance, Smith et al. [18] propose a blockchain-based architecture tailored for IoT systems, emphasizing its potential for real-time data processing and secure communication in distributed environments. Their findings align with our approach, particularly in addressing the challenges of scalability and interoperability in IoT–blockchain integration.

Beyond theoretical frameworks, real-world implementations further demonstrate blockchain’s transformative impact across various domains. In smart cities, Dubai’s blockchain-powered governance system secures transactions for urban planning, traffic management, and renewable energy trading, while Singapore leverages blockchain for decentralized water management, ensuring real-time monitoring and automated distribution [17,19]. The agricultural sector benefits from blockchain platforms like AgriDigital in Australia and IBM Food Trust, where IoT-enabled sensors track grain supply chains and farm conditions, ensuring transparency and automating payments through smart contracts [15,20]. Healthcare applications such as MedRec decentralize patient records for enhanced security and interoperability, while PharmaLedger employs blockchain and IoT sensors to prevent counterfeit drugs and ensure regulatory compliance [16,19]. In industrial automation, Bosch integrates blockchain with Ethereum smart contracts for secure machine identity management, and Siemens deploys blockchain-based IoT networks for autonomous manufacturing, eliminating single points of failure and improving efficiency [19,20]. These case studies illustrate how blockchain, combined with IoT, not only enhances security and trust but also enables automation, real-time analytics, and decentralized decision-making across diverse industries.

### 2.3. Smart Contracts

The authors of [21] explore the advancement, issues, and potential future directions of smart contracts in blockchain technology. The study highlights the significance of blockchain in securely recording transactions and presents smart contracts as a key innovation in the blockchain ecosystem. Moreover, ref. [21] emphasizes the potential uses of smart contracts and blockchain technology in various sectors, including healthcare, supply chain management, and energy. Additionally, the study addresses the challenges faced by smart contracts, such as limitations in data processing capabilities, security vulnerabilities, and legal and privacy concerns. The authors of [21] also touch upon the potential challenges and legal considerations for smart contracts and the measures needed for their future development.

Reference [22] explores the potential of utilizing blockchains and smart contracts in the IoT domain. It first explains the concept of blockchains and smart contracts and discusses their application in the IoT sector. The study highlights how the blockchain–IoT combination can facilitate the sharing of services and resources, leading to the creation of a marketplace of services between devices. This concept serves as a blueprint for establishing an external entity to operate blockchain within the IoT context. The authors of [22] also emphasize how this combination can automate existing time-consuming workflows in a cryptographically verifiable manner. Furthermore, ref. [22] identifies several issues that should be considered before deploying a blockchain network in an IoT setting, ranging from transactional privacy to the expected value of digitized assets traded on the network. The paper concludes that the blockchain–IoT combination has the potential to drive significant transformations across various industries, paving the way for new business models and innovative distributed applications.

The integration of IoT technology with smart contracts on blockchain platforms is opening doors to innovative solutions across various industries. Smart contracts, which are self-executing agreements stored on a blockchain, act as automated facilitators of predefined rules and conditions, adding a new layer of trust and efficiency to IoT applications.

Integrating smart contracts with IoT devices enables secure and automated decision-making based on predefined conditions. Data collected by sensors can trigger the execution of smart contracts, eliminating the need for manual intervention and potential human error. This ensures transparency and reduces the risk of fraud in data-driven processes [16].

This synergy is already leading to real-world applications. In supply chain management, smart contracts can automatically trigger payments upon the successful delivery of goods, confirmed by IoT sensors, streamlining financial transactions and enhancing trust between trading partners [20]. Similarly, in the energy sector, smart grids equipped with IoT sensors and smart contracts can facilitate automated energy trading based on real-time energy consumption data, enabling peer-to-peer transactions and optimized resource utilization [19].

## 3. IoT Operator Definition

Within the TpM+ framework, the IoT Operator emerges as a pivotal entity, weaving together data guardianship, contract enforcement, and conflict resolution to foster a cohesive and trustworthy ecosystem.

Acting as a trusted intermediary, the IoT Operator orchestrates a multifaceted operation, ensuring the responsible stewardship of information and the smooth functioning of the interconnected system. It serves as a custody caretaker, contract enforcer, and fair mediator, all integrated into a single entity. This is the essence of the IoT Operator within the TpM+.

One of the fundamental challenges faced by companies in the burgeoning landscape of the IoT is the proactive monitoring and historical tracking of connected devices, often referred to as “Things”. Traditional solutions suffer from limitations, and these include the following:

- Impractically complex: Setting up intricate individual tracking systems for each device can be resource-intensive and unwieldy, hindering scalability and efficiency.
- Data silos: Tracking data are often confined within specific applications or device ecosystems, lacking a centralized, holistic view that enables comprehensive analysis and informed decision-making.

Herein lies the crucial role of the IoT Operator within the TpM+ framework. It acts as a meticulous custodian, overseeing the extensive flow of monitoring data generated by interconnected devices. Think of it as a vigilant watchtower, constantly tracking the location and historical movements of each “Thing” within the system.

Beyond mere data vaulting, the Operator crafts a transparent framework of accountability, weaving together location and historical data into a readily accessible web of knowledge. Employing sophisticated data aggregation and visualization techniques, the Operator empowers authorized stakeholders with intuitive dashboards and insightful reports. Thus, the IoT Operator is not merely a passive data custodian but an active enforcer of data integrity, accessibility, and accountability, enabling companies to navigate the complexities of interconnected systems with confidence and precision [4].

The Operator also acts as the impartial interpreter of smart contracts, with its algorithms meticulously analyzing each clause and provision. It navigates the complexities of conditional statements, triggers, and execution protocols with unwavering precision, ensuring that every interaction aligns with the contract’s intent. This rigorous interpretation minimizes ambiguity and fosters trust among stakeholders, knowing that the Operator will uphold the agreements without bias or favoritism.

The Operator wields the power of cryptographic logic [23], acting as a bridge between the complex language of smart contracts and the human experience of disagreement. Its algorithms meticulously analyze the contractual stipulations in relation to the data it safeguards, identifying relevant clauses and provisions that inform potential solutions. This transparent interpretation empowers stakeholders to grasp the legal and digital landscape of the conflict, fostering collaboration and informed decision-making.

**Figure 1:**
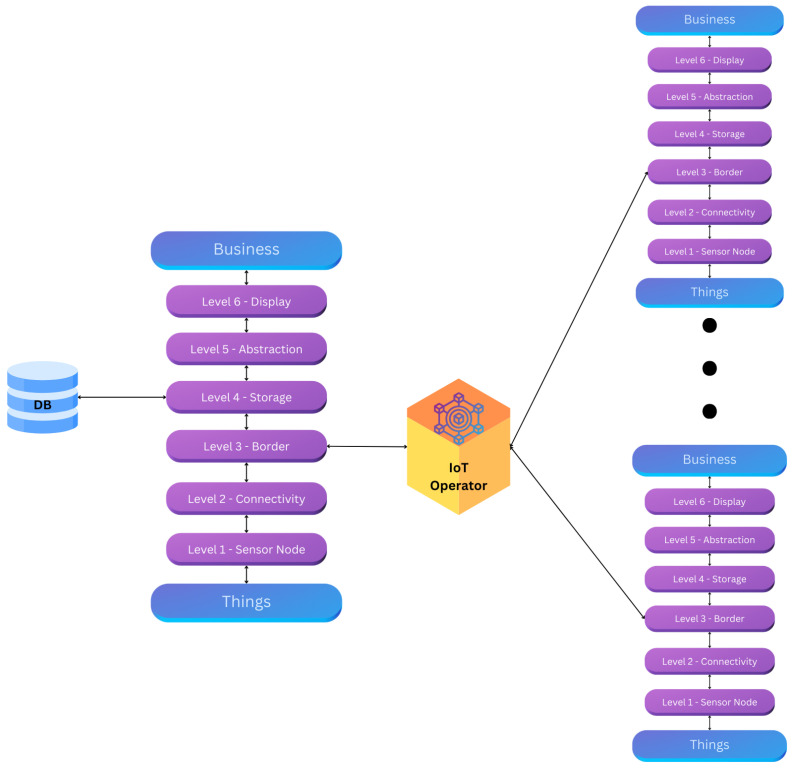
IoT Operator roles to connect with the border of each singular TpM.

Thus, the final IoT reference model, based on the work of [8], is structured to incorporate these essential functions, as illustrated in Figure 1.

### 3.1. Custody Caretaker

To guarantee irrevocable data custody within the TpM+ framework, the proposal introduces the IoT Operator as a dedicated custodial entity. This role entails meticulously safeguarding every transaction and data point generated within the interconnected landscape, ensuring their immutability [24].

Recognizing the critical need for inherent trust and transparency, the IoT Operator leverages a blockchain-based approach. This technology aligns perfectly with the TpM+ ethos due to its core strengths: decentralization, tamper-proof record-keeping, and verifiable audit trails.

However, TpM+ departs from traditional blockchains by eschewing a globally distributed network of miners. Instead, it employs a hybrid consensus mechanism characterized by localized execution [24] (which occurs at Level 3—border) and centralized validation. While the foundation remains decentralized, with geographically dispersed nodes responsible for data validation, the consensus process itself is centralized within the IoT Operator. These nodes are meticulously identified in Phase 2 and deployed in Phase 3. Whenever a user at the Operational level initiates a state change, the designated node assumes the role of data custodian, making the user accountable for the transaction and captured measurements:

- Efficiency: Eliminating the need for global participation in the consensus process can significantly improve processing speed and scalability, especially in the dynamic environment of the IoT.
- Scalability: Centralized consensus within clusters streamlines the process of implementing changes or resolving disputes, leading to faster and more efficient governance.
- Transparency: Each node contributes to the consensus process, creating a transparent and verifiable audit trail for every transaction and data point. This empowers stakeholders with clear insights into the system’s operations.

The audit trail framework and the application of blockchain within the IoT Operator are illustrated in Figure 1.

Figure 1 illustrates the audit trail created when utilizing the blockchain approach within the IoT Operator. Whenever one of the “Things” changes state, its designated guardian is updated. Additionally, the figure highlights the scalability of blockchain, demonstrating how the system can accommodate an increasing number of “Things” as needed.

In essence, the TpM+ framework proposes a tailored blockchain implementation that leverages the inherent strengths of decentralization while addressing the specific needs of the interconnected IoT environment. By establishing the IoT Operator as a dedicated custodian and employing a centralized consensus mechanism, TpM+ ensures secure and irrevocable data custody, which is fundamental to trustworthy operations within the interconnected world.

### 3.2. Contract Enforcer

The TpM+ framework’s embrace of blockchain technology promises an environment of immutable accountability for interconnected devices. Every whisper of data, every action taken, and every pulse of the system are inscribed onto the transparent ledger, fostering trust and predictability. However, within this tapestry of accountability emerges a crucial query: how does it enforce the smart contracts—the digital pacts—that bind the Operational-level entities?

**Figure 2:**
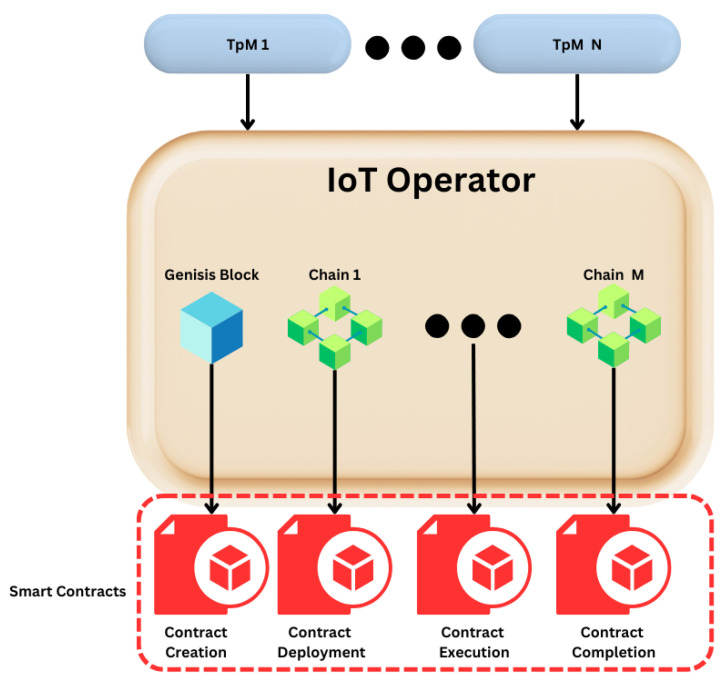
Smart contracts in conjunction with the IoT Operator.

This question stands as the core challenge at the heart of TpM+ contract enforcement. Unlike traditional agreements that rely on centralized authorities and legal frameworks, smart contracts self-execute based on pre-programmed conditions. They eliminate the need for intermediaries, and they can be delegated by the IoT Operator. Figure 2 below illustrates the process of creating a smart contract within the IoT Operator.

In Figure 2, we can observe the four key steps of a smart contract within the IoT ecosystem: creation, deployment, execution, and completion:

- Creation: The smart contract is created when someone from the Operational level agrees to work under the IoT Solution.
- Deployment: The smart contract is immediately deployed when a user from the Operational level changes the state of one of the “Things” that is being monitored.
- Execution: Whenever one of the rules established during contract creation is broken, such as unauthorized changes in the system or a malfunction, the system will take the necessary steps to deactivate the user or enforce any other predefined action within the contract.
- Completion: The contract is complete when the “Thing” reaches its final destination or when a user exits the system.

This autonomous enforcement, facilitated by the IoT Operator and empowered by blockchain’s immutability, marks a significant advancement in contract management. The complexities of enforcement are replaced with the reliability of automated execution and decentralized governance. While challenges remain, the path forward is increasingly clear, driven by the potential of smart contracts to usher in a new era of trust and accountability within the interconnected world.

### 3.3. Mediator

The embrace of smart contracts and blockchain technology within the TpM+ framework promises a landscape of immutable accountability. However, this digital infrastructure also presents the potential for disagreements, particularly within the context of complex interactions between the Operational and Executive levels. Here, the IoT Operator emerges as a vital entity—not merely a facilitator of agreements but a custodian of resolution—equipped to navigate these inevitable contentions with transparency and impartiality.

The Operator’s role in mitigating disagreements hinges on two key pillars:

- The immutable audit trail: The blockchain ledger serves as an irrefutable record of every action and data point within the system. This transparent audit trail provides an unbiased foundation for resolving disputes. Unlike traditional contracts, which are often ambiguous, smart contracts inscribed on the blockchain offer a crystal-clear narrative, eliminating the potential for misinterpretations or manipulation. Stakeholders at both the Operational and Executive levels can access this verifiable record, empowering them to present their arguments based on objective data.
- A neutral ground for resolution: The decentralized nature of the TpM+ framework, coupled with the Operator’s blockchain-powered neutrality, fosters a level playing field for dispute resolution. Unlike traditional legal systems that rely on centralized authorities, the Operator facilitates a participatory process where both the Operational and Executive levels can share their perspectives. This collaborative approach fosters mutual understanding and trust, paving the way for informed and equitable solutions.

Furthermore, the Operator’s role extends beyond simply presenting the facts and facilitating communication. Its advanced algorithms can analyze the historical data stored on the blockchain, identifying patterns and trends that might shed light on the nature of the disagreement. This data-driven insight can empower stakeholders to reach evidence-based resolutions, ensuring that outcomes are not only fair but also informed by the totality of the situation.

To further enhance the efficiency and scalability of the Operator’s functions, the integration of hybrid consensus mechanisms characterized by localized execution is proposed. These mechanisms combine elements of multiple consensus protocols to optimize transaction validation and block finalization while ensuring security, decentralization, and low latencies. Unlike monolithic consensus models, a hybrid approach allows the IoT Operator to balance efficiency and trust by leveraging different consensus layers for various transaction types.

For example, Polkadot employs a hybrid consensus model where block production and finality are handled by separate mechanisms. The BABE (blind assignment for blockchain extension) protocol is used for probabilistic block production, ensuring a continuous transaction flow with minimal latency. In parallel, the GRANDPA (GHOST-based Recursive ANcestor Deriving Prefix Agreement) finality mechanism is employed to ensure global consistency and security [25]. This separation allows for high transaction throughput while maintaining robust Byzantine Fault Tolerance (BFT). Similarly, hybrid consensus approaches that integrate Proof-of-Work (PoW) and Proof-of-Stake (PoS) have been studied to enhance blockchain resilience. PoW mechanisms are leveraged for initial transaction validation, ensuring trust and resistance to Sybil attacks, while PoS mechanisms finalize blocks efficiently by selecting validators based on their stake and reputation [26].

Within the IoT Operator, a hybrid consensus model can be structured in three distinct layers:

- Edge-level validation: IoT devices (Level 1) and the local border (Level 3) use a lightweight Proof-of-Authority (PoA) model for immediate transaction validation, reducing computational overhead and minimizing energy consumption.
- Cluster-level aggregation: Validated transactions are aggregated into proto-blocks, following a Delegated Proof-of-Stake (DPoS) or leader-election model to optimize efficiency while ensuring trustworthiness.
- Global finalization: The IoT Operator uses an asynchronous BFT mechanism, similarly to GRANDPA, to finalize transactions across interconnected IoT networks, ensuring immutability and global consensus without introducing unnecessary delays.

This layered hybrid approach allows localized execution for real-time responsiveness while ensuring network-wide consistency and trust through finalization mechanisms. By adopting this model, the IoT Operator enhances transaction throughput, minimizes latency, and prevents bottlenecks inherent in traditional blockchain implementations. Additionally, the hybrid model mitigates centralization risks, as different consensus layers distribute responsibility dynamically, preventing single points of failure and increasing system resilience.

In conclusion, the potential entailment for disagreements within the TpM+ framework is not a threat but rather an opportunity to showcase the strengths of this innovative system. The IoT Operator, equipped with the irrefutable power of the blockchain and a commitment to neutrality, stands ready to guide stakeholders towards amicable resolutions, fostering a climate of trust and collaboration within the interconnected world.

## 4. TpM+ Proposal

### 4.1. IoT Operator as Common Denominator

As [6] aptly highlights, information systems can be conceptualized as specialized subtypes of work systems, where the latter encompasses objects requiring development or integration through a structured methodology. In the context of IoT applications, this framework takes on particular significance. As [7] observes, IoT inherently involves the integration of multiple subsystems, painting a rich tapestry of interconnected work systems. However, this heterogeneity often presents a challenge: a lack of common ground for information exchange, leading to a lack of transparency among these systems. This acknowledgment underscores the critical necessity for a trusted external entity, an “IoT Operator”, capable of cultivating transparency and fostering seamless collaboration across the diverse work systems that comprise IoT ecosystems.

The initial phase of the TpM plays a crucial role in defining the “IoT Operator” and its function within the business context. This necessitates the rigorous application of the TpM-IoT Canvas, with a primary focus on both the business goals and the characteristics of the product or service. The identified “IoT Operator” must then align its activities with the overarching business objectives and strategically identify key processes that would enhance operational transparency through data-driven insights.

### 4.2. Phase 1

**Figure 3:**
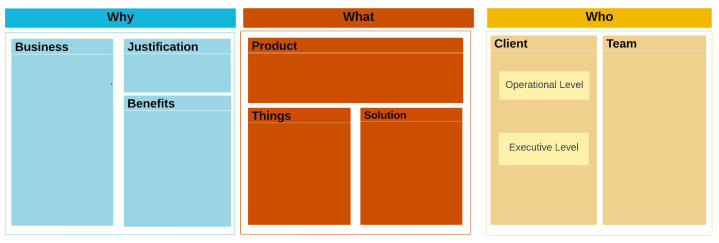
TpM-IoT Canvas for TpM+.

The TpM highlights the multifaceted benefits of the usage of TpM-IoT Canvas [6,7], emphasizing its efficacy in not only generating comprehensive reports but also in clearly delineating the business context. Figure 3 serves as a visual representation of the key points required for a continuous IoT solution. In the “Client” entity, as an extension to the TpM-IoT Canvas, we break the IoT solution down into two different levels: the “Executive” and the “Operational” Level. These levels are defined as follows:

- Executive level: This stratum assumes responsibility for pivotal decision-making, strategic directives, and the overarching vision of the solution. Emphasizing its critical role, this level dictates the solution’s direction and success, handling tasks such as resource allocation, risk management, and performance evaluation.
- Operational level: This level encapsulates the actions and mechanisms that constitute the operational facet of the IoT solution. Common tasks at this level include operating the “Things”, performing data analysis, and acquiring data.

Clear and effective communication between the Executive and Operational levels is essential, as it defines their intricate relationship and collaboration within the IoT domain. These interconnections necessitate a meticulous definition of responsibilities for all participants in the solution, including devices, data flows, and processes within the system. Recognizing the complexities and potential for misalignment within such an ecosystem, we propose the establishment of an independent “IoT Operator”. This dedicated entity acts as a central facilitator of transparency, accountability, and informed decision-making between the aforementioned levels. During the initial phase of TpM+, both levels formalize their commitments through a smart contract mediated by the “IoT Operator”. Leveraging blockchain technology ensures verifiable compliance and a trusted data trail, further solidifying trust and accountability within the system.

With TpM+, the first phase becomes a collaborative process, where, at each iteration, the TpM IoT Canvas is completed:

- Business: Defines the enterprise’s area of expertise, core activity, and the domain in which the proposed solution operates, along with its relevance to the company’s activities.
- Justification: Provides the rationale for implementing the IoT solution, emphasizing its alignment with the company’s core objectives.
- Benefits: Outlines the tangible and intangible value propositions expected from the project, which justify the solution’s development.
- Product: Defines the solution’s core elements, whether in the form of hardware, software platforms, system integration, or service platforms.
- Things: Lists the elements interacting with the solution and specifies what should be measured, controlled, or quantified for each “Thing”.
- Solution: Encompasses all solution requirements, reflecting business rules and justifications. This section also serves as the foundation for Phase 2.
- Client: Represents individuals or organizations leveraging IoT technologies to enhance their operations, products, or services:
  – Operational: Describes how individuals will operate the solution daily.
  – Executive: Defines the expectations of business leaders and decision-makers regarding the IoT solution’s impact.
- Team: Details the development team responsible for building and deploying the IoT solution.

### 4.3. Phase 2

During the second phase of TpM+, the iterative process of “Gathering Requirements”, the addition of the “IoT Operator” strengthens the framework while preserving its core structure [27]. The second phase remains a top-down approach [8], but it is process-oriented and guided by the TpM-IoT Canvas established in Phase 1, where the following is the case:

- Level 6—display: Defines how information is presented to the Executive level.
- Level 5—abstraction: Covers general data processing and decision-making mechanisms. It is also the level where the IoT contract is located.
- Level 4—storage: Specifies which data points should be stored and includes historical data records.
- Level 3—border: Acts as the bridge between local and external networks. It also defines processing and storage functions for local data and establishes the connection between the Operational and Executive levels.
- Level 2—connectivity: Determines the communication protocols linking the “Things” to the border.
- Level 1—sensor/actuator: Consists of devices responsible for monitoring, controlling, and operating the “Things”.

A significant advancement occurs at Level 3, the “border” layer, where it extends into both local and cloud environments to accommodate Executive and Operational requirements. The cloud border consolidates data from various sensors and devices (Level 1) through multiple communication protocols (Level 2). This unique characteristic of TpM+, where Level 3 effectively acts as an umbrella for the lower levels, makes it the ideal integration point for the IoT Operator.

**Figure 4:**
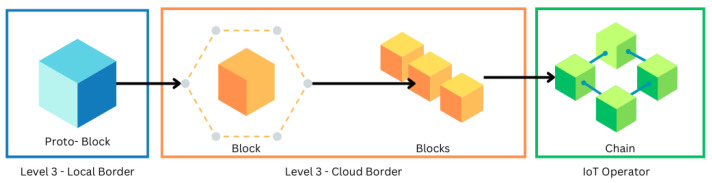
Block creation on TpM+.

This functionality is further enhanced by the “IoT Operator” at Level 3. Here, the local border generates a “proto-block”, a data packet containing identification details. The proto-block is then sent to the cloud border for validation. Once validated, it joins other verified blocks and is ultimately transmitted to the IoT Operator, forming a secure and transparent chain of data. This process is illustrated in Figure 4.

Phase 2 focuses on clearly defining the IoT Operator’s role, including its data monitoring functions, preferred data formats, and contributions to system transparency and accountability.

### 4.4. Phase 3

The third phase of TpM+ [7,27] focuses on the practical implementation of the IoT solution. This phase follows a bottom-up approach, where each level (1, 2, and parts of 3) is implemented with careful consideration of the specific “Things” being monitored. This ensures that each component is tailored to the system’s unique requirements.

During the second step of implementing Level 3, the “cloud border” must account for the communication protocols and data formats of each local border and the “Things” they manage. This complexity underscores the need for the IoT Operator as a central authority for managing custody and oversight.

At Level 4 (storage), the IoT Operator integrates a blockchain-centric approach for securely recording transactions. While relational and NoSQL databases may be used at this level, blockchain’s inherent strengths—immutability and speed [4]—establish a transparent, verifiable record of custody for the Executive level.

**Figure 5:**
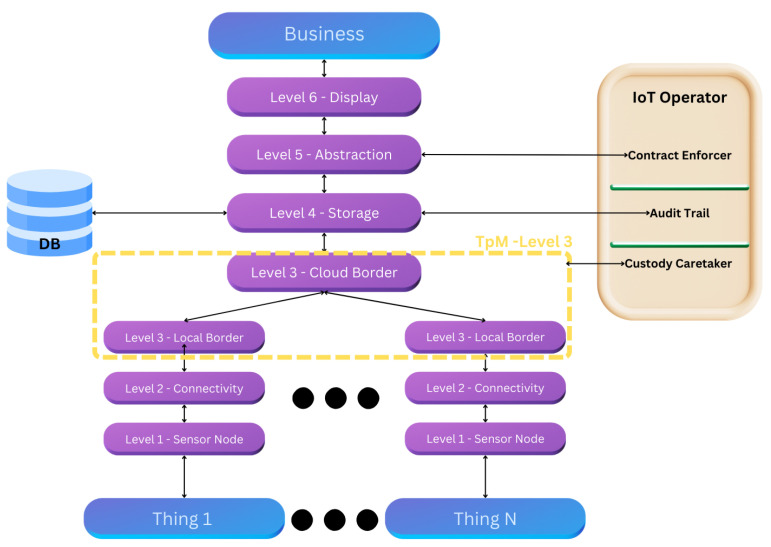
TpM+ third-phase reference model.

Phase 3 culminates in Level 6 (display), where data points are visualized in an accessible manner for decision-makers, completing the IoT solution’s life cycle. This process is depicted in Figure 5.

## 5. Results

### 5.1. PoC

This section explores the practical application of the concepts discussed in this paper. Proof of Concept (PoC) exercises have been employed to bridge the gap between theoretical possibilities and real-world demonstrations of feasibility and effectiveness. Through this PoC, the solution aimed to not only validate the technical viability of our proposed solutions but also gain valuable insights into their real-world performance and potential challenges.

Located in Campinas, Brazil, the Eldorado Research Institute, a renowned non-profit organization, fosters advancements in research, development, and innovation. However, their testing equipment, while accessible to diverse sectors, suffers from inefficiencies due to a reliance on paper-based control systems. This outdated process often results in missing, misplaced, or difficult-to-locate equipment, leading to wasted time and resources.

To address this issue, an IoT solution guided by TpM+ was implemented, and it was designed to enhance efficiency, transparency, auditability, and security in the management of testing equipment. This solution leverages hardware-based security features embedded within the equipment itself, coupled with a cloud-based platform for centralized control and visibility.

#### 5.1.1. Phase 1

Through intensive analysis, it is possible to solidify our understanding of the players and business landscape, visualizing it effectively on the TpM-IoT-Canvas seen in Figure 3. In this figure, the following is noted:

- Business: The primary goal is to implement a solution for localizing and monitoring testing hardware within the organization.
- Justification: Testing hardware is often misplaced, missing, or difficult to locate, leading to inefficiencies and downtime in the development workflow.
- Benefits: The proposed system delivers time-saving capabilities, enhances accountability, and improves inventory management by providing real-time tracking and access control. Additionally, it ensures auditability, non-repudiation, and transparency regarding equipment ownership.
- Product: A testing hardware monitoring tool, which integrates IoT-enabled sensors and a web-based management interface to streamline equipment tracking.
- Things: The primary assets being monitored include various testing hardware used by the development team.
- Solution: The system involves embedding IoT sensors on both the testing hardware and in designated testing rooms where the equipment can be taken. The collected data will be processed and managed via a web-based application, allowing real-time equipment tracking and generating reports on hardware usage and availability.
- Client: The solution is developed for the Eldorado Institute, aiming to enhance their operational workflow:
  – Operational level: Used by the Eldorado Development Team to efficiently locate and manage testing hardware.
  – Executive level: Utilized by managers and directors for oversight, reporting, and strategic decision-making.
- Team: The development is carried out by the Unicamp Development Team in the Wistek Lab, consisting of the following:
  – Three undergraduate students, responsible for implementation and system integration.
  – Two PhD students, overseeing advanced development and research-driven optimizations.
  – A titular researcher, supervising the project and ensuring alignment with research objectives.

#### 5.1.2. Phase 2

**Figure 6:**
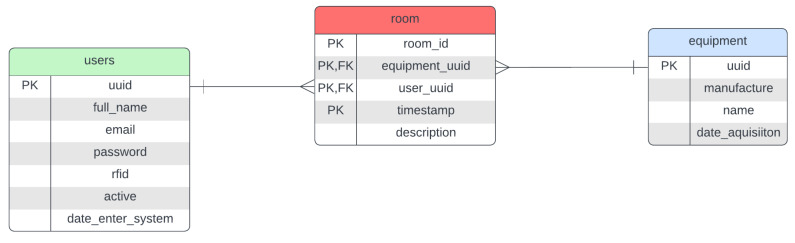
Entity relationship diagram.

Building upon the identified business landscape, the solution employed delved deeper in Phase 2 to collect specific requirements for sensor data and web application functionalities, laying the groundwork for the upcoming design and development stages. This is described bellow:

- Level 6—display: A web application that can be executed in any browser or phone.
- Level 5—abstraction: Needs authentication for the web app and needs an export function and the real-time monitoring of where the hardware has been.
- Level 4—storage: Structured and fast search data. To accomplish this, data modeling was created based on the entity relationship (ER) diagram; it is widely used in describing database structures and visually represents entities, their attributes, and the relationships between them to facilitate database design and understanding. Refer to [28], as shown in Figure 6. In this Figure, we can see that there should always be someone responsible for equipment in a particular testing room.
- Level 3—border: Needs an Internet interface.
- Level 2—connectivity: Can be connected via cable.
- Level 1—sensor/actuator: Small sensors that can be embedded on the hardware and on the rooms.

#### 5.1.3. IoT Operator—Phase 2

During Phase 2, the IoT Operator is integrated into Levels 3, 4, and 5 to facilitate transparent equipment usage tracking and enforce accountability. Here is how it works:

- Data block: Each data block stores information about the user, room, equipment, and timestamp.
- Thing identification: A “Thing” is identified by a combination of the user ID, equipment ID, and the room it is used in.
- Smart contract activation: A smart contract automatically triggers if a user uses a piece of equipment for more than 24 h, potentially flagging unauthorized extended use.
- Equipment responsibility: A designated user is assigned responsibility for all equipment, ensuring clear accountability.

#### 5.1.4. Phase 3

**Figure 7:**
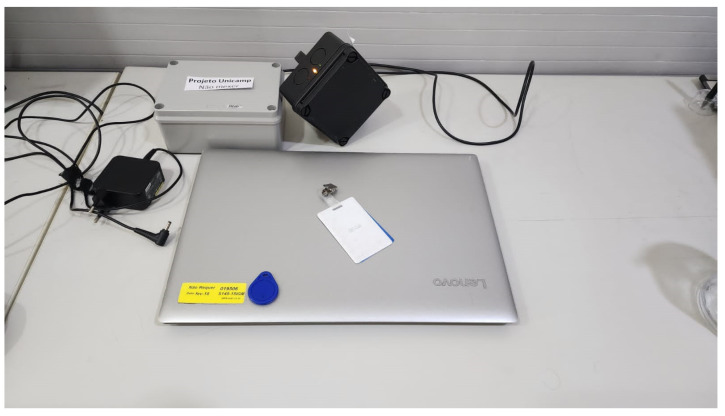
Implementation of PoC Level 1.

**Figure 8:**
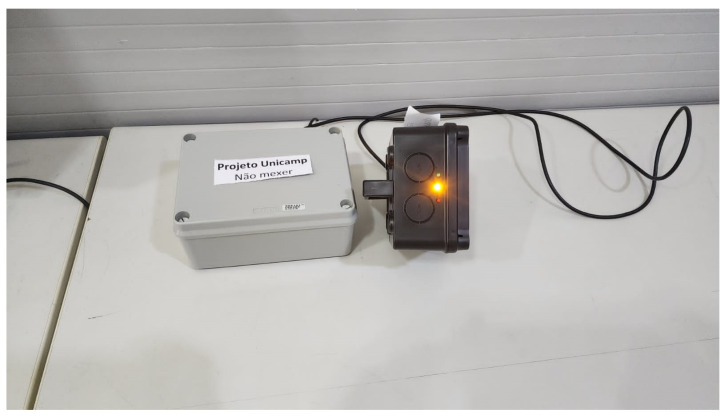
Implementation of PoC Level 3.

**Figure 9:**
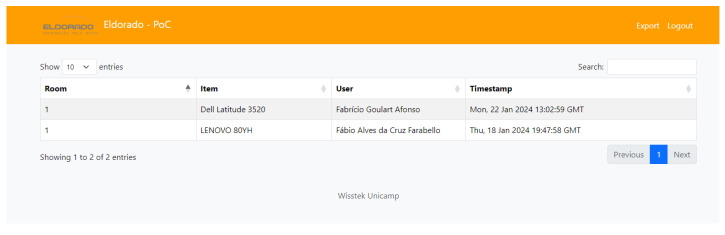
Implementation of PoC Level 5 and 6.

During Phase 3, the technologies are chosen and applied as follows:

- Level 1—sensor/actuator: RFID in both equipment and utilizing the users cards for credentials, as in Figure 7.
- Level 2—connectivity: Cable.
- Level 3—border: Raspberry Pi 5 with Python 3.12, illustrated in Figure 8.
- Level 4—storage: SQL.
- Level 5—abstraction: Python, using Flask.
- Level 6—display: html, Javascript, and CSS, as shown in Figure 9.

**Figure 10:**
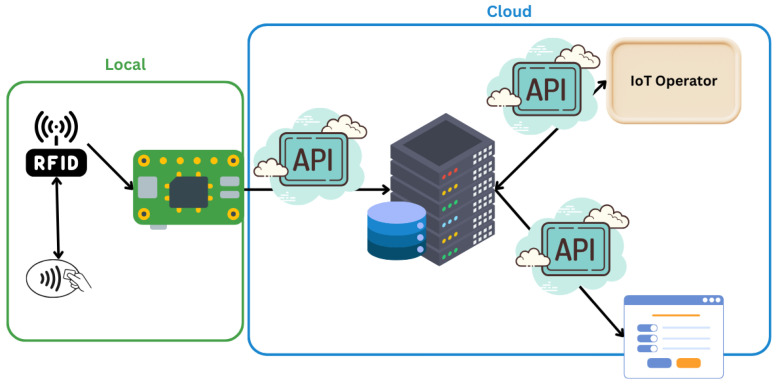
IoT solution architecture illustrating data flow from local devices to cloud-based services and the IoT Operator.

To illustrate the implementation, the diagram Figure 10 below shows the architecture of an IoT solution structured into multiple levels. This structured approach ensures seamless data flow, storage, and accessibility within the IoT ecosystem in conjunction with the IoT Operator.

#### 5.1.5. IoT Operator—Phase 3

Phase 3 focuses on implementing a secure IoT Operator application. This application utilizes the Flask framework and leverages REST APIs for secure interaction with the blockchain network. Employing HTTPS communication and requiring authentication through JSON Web Tokens (JWTs) adhere to best security practices, ensuring the confidentiality and integrity of data in transit.

The decision to leverage REST APIs stems from their inherent advantages, including the following:

- Agility and simplicity: REST APIs are characterized by a straightforward design and intuitive nature, fostering rapid development and efficient utilization.
- Flexibility and scalability: The inherent stateless nature and resource-oriented architecture of REST APIs enable their adaptability to diverse use cases and seamless scaling to accommodate large-scale data workloads.
- Ease of integration: Compatibility with established protocols and widespread adoption across various systems facilitate the integration of REST APIs with diverse technologies, such as smart contracts in this instance.

To ensure scalability, low latency, and security in IoT–blockchain integration, the IoT Operator created in Python employs a hybrid consensus mechanism. This model consists of three layers:

- Edge-level validation: IoT devices use Proof-of-Authority (PoA) for fast and lightweight transaction validation.
- Cluster-level aggregation: Delegated nodes employ delegated Proof-of-Stake (DPoS) to aggregate validated transactions into proto-blocks.
- Global finalization: A Byzantine Fault Tolerant (BFT) mechanism ensures cross-network consensus and immutability.

Furthermore, to safeguard data at rest, the implementation incorporates AES encryption, leveraging private keys as documented in [29]. This encryption mechanism provides an additional layer of security, protecting sensitive information stored within the system, providing search agility, and enforcing contracts.

### 5.2. PoC Results

To evaluate the success of the proposed IoT solution and its impact on Total Productive Maintenance as an employed metric, the time to find equipment was considered: measurement of the average time required to locate a specific equipment piece within the operational environment. A significant reduction in this time indicates improved efficiency and productivity. Table 1 shows the difference in time before the implementation of the IoT solution based on TpM+ and after it.

The presented table clearly demonstrates the substantial improvement achieved with the IoT solution. By reducing the time to find equipment from 2 h to 5 min, the solution delivered a remarkable 95.83% reduction. This translates to significant benefits, such as operational efficiency (reduced downtime due to quicker equipment availability, leading to increased production output), improved maintenance responsiveness (faster identification of equipment issues, enabling timely intervention and preventive maintenance), and reduced labor costs (less time spent searching for equipment translates to more efficient use of personnel resources).

Beyond efficiency, a fundamental goal of this study is to enhance transparency within IoT-driven environments. Traditional systems often lack visibility into real-time equipment status and historical interactions, making it difficult to verify actions, ensure compliance, or track unauthorized modifications. With the integration of blockchain-backed audit trails, every event, status change, or interaction is immutably recorded, ensuring tamper-proof and verifiable data storage. This approach eliminates ambiguities in system records, allowing stakeholders to trust the integrity of operational data. The qualitative benefits of this enhanced transparency include greater trust between departments, seamless compliance with industry standards, and more informed decision-making based on immutable records rather than subjective interpretations.

Furthermore, the IoT solution strengthens accountability by ensuring that all actions, whether they involve asset transfers, maintenance activities, or configuration changes, are irrevocably recorded and linked to responsible entities. Through a structured identity management system, each transaction is cryptographically signed, preventing unauthorized interventions and enabling a clear traceability path for all activities. This not only helps in assigning responsibility but also ensures that errors or misconduct can be efficiently identified and rectified. The qualitative benefits of this enhanced accountability include a shift from reactive issue resolution to proactive governance, reduced internal disputes, and a strengthened culture of responsibility within the organization.

**Figure 11:**
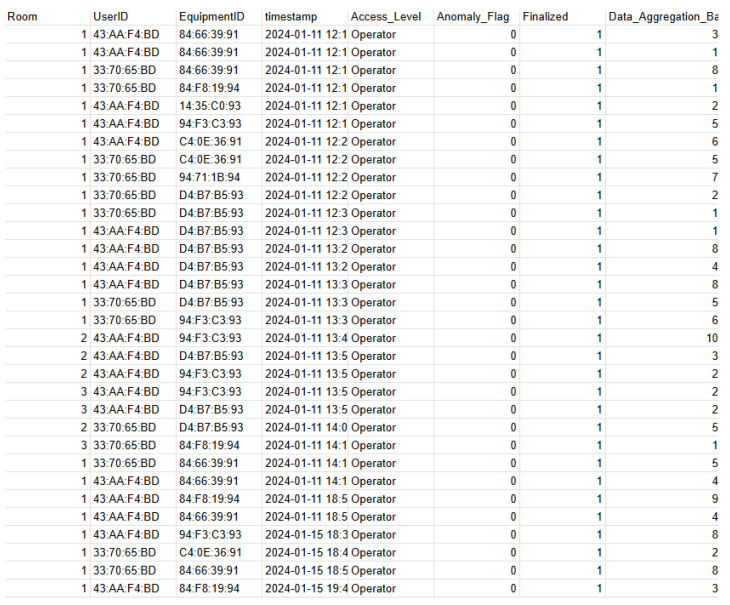
Snippet of the IoT data chain: from sensor capture to immutable blockchain records.

Figure 11 visually represents the IoT events (a user taking custody of equipment), illustrating the movement of users, equipment interactions, and the structured data validation process:

- The data in the chain record 87 access events across three unique rooms, involving two different equipment units and three users.
- All access interactions are associated with two access levels: “Operator” and “Admin”.
- Every event is fully finalized, ensuring that all transactions are completed and validated, meaning that there is no violation of the contracts.

- Room 1 recorded the highest number of interactions, with Equipment “84:66:39:91” being accessed the most frequently.
- Each transaction is associated with a unique UserID, ensuring traceability and accountability.
- The IoT chain enforces access monitoring, ensuring that all interactions are securely recorded without anomalies.

By integrating transparency, accountability, and operational efficiency, the TpM+ framework provides a robust foundation for intelligent, data-driven decision-making within industrial environments. The study demonstrates that automation and decentralized validation mechanisms not only improve efficiency but also instill trust in data integrity and compliance adherence. Moreover, it was observed that Operator users, who are aware of the accountability mechanisms in place, exhibited more responsible behavior when handling equipment. The immutable logging of interactions fostered an environment where adherence to best practices became the norm, reducing equipment misuse and untracked modifications. This behavioral shift underscores the effectiveness of transparency in promoting operational discipline and responsible resource management.

Furthermore, the structured audit trials not only serve as a compliance mechanism but also enhance stakeholder trust by providing an indisputable record of all interactions. The integration of blockchain-backed validation ensures that decision-making processes rely on verifiable, tamper-proof data rather than subjective reports. Future work may explore extending this model into broader applications, such as supply chain logistics and real-time asset verification in large-scale industrial ecosystems. Ultimately, the proposed solution establishes a scalable, resilient, and accountable IoT governance framework, ensuring operational excellence in interconnected environments.

## 6. Conclusions

This paper addressed the challenges of heterogeneity within the IoT landscape, characterized by diverse standards and vendor-specific solutions that hinder resource gathering and implementation. The concept of standardized IoT solutions proposed by TpM was explored, and the TpM+ model, an extension of TpM that incorporates an external trustworthy “IoT Operator”, was introduced.

This IoT Operator leverages blockchain technology and smart contracts to function as a custody caretaker, contract enforcer, and mediator, ensuring transparency, security, and trust within the IoT ecosystem. To evaluate the effectiveness of TpM+, a full IoT solution was implemented at the Eldorado Institute, focusing on reducing equipment search time.

The results were significant, demonstrating a remarkable 95.83% reduction in equipment search time. This achievement validates the success of the TpM+ model in delivering faster equipment location, which can be directly attributed to the enhanced custody and accountability mechanisms enabled by the IoT Operator. Beyond efficiency, the integration of accountability and transparency further strengthens the system’s reliability. The immutable tracking of interactions ensures that all equipment movements are fully auditable, reducing unauthorized modifications and promoting responsible usage. Additionally, the presence of structured audit trails fosters greater stakeholder trust, as decision-making is based on verifiable, tamper-proof data rather than subjective reports. Future studies could involve partnering with larger organizations across various industries to test the scalability of TpM+ and identify potential modifications needed for broader deployment.

This paper not only highlights the practical benefits of TpM+ but also advances the broader goal of IoT standardization by proposing a framework that enhances interoperability and reduces vendor lock-in.

In conclusion, by establishing standardized APIs and data exchange protocols, TpM+ has the potential to serve as a central hub for real-time data handling, facilitating data-driven decision-making and performance optimization across various enterprise systems.

## Figures and Tables

**Table 1:** IoT solution’s average time to find equipment comparison.

Application	Time	Time %
Without IoT Solution	2 h	100%
IoT Solution	5 min	4.167%

## Data Availability

Data are contained within the article.

## References

[1] Vaiyapuri T., Parvathy V.S., Manikandan V., Krishnaraj N., Gupta D., Shankar K. (2021). A Novel Hybrid Optimization for Cluster-Based Routing Protocol in Information-Centric Wireless Sensor Networks for IoT Based Mobile Edge Computing. Wirel. Pers. Commun..

[2] Ferreira L.C.B., Chaves P.R., Assumpção R.M., Branquinho O.C., Fruett F., Cardieri P. (2022). The three-phase methodology for iot project development. Internet Things.

[3] Groover M.P. (2016). Automation, Production Systems, and Computer-Integrated Manufacturing.

[4] Kamath R. (2018). Food traceability on blockchain: Walmart’s pork and mango pilots with IBM. J. Br. Blockchain Assoc..

[5] Salman T., Jain R. (2015). Networking protocols and standards for internet of things. Internet Things Data Anal. Handb..

[6] da Assumpção R.M. (2023). Didactic Strategy for Iot Education and Integration of Blockchain and Smart Contracts with the Use of Tpm. Ph.D. Thesis.

[7] Ferreira L.C.B., Yamaguti R., Branquinho O.C., Cardieri P. (2022). A TpM-based collaborative system to teach IoT. Comput. Appl. Eng. Educ..

[8] Déo A.L.B. (2018). Proposta de Um Modelo de Referência Open Source para IoT.

[9] Haber S., Stornetta W.S. (1991). How to Time-Stamp a Digital Document.

[10] Becker G. (2008). Merkle Signature Schemes, Merkle Trees and Their Cryptanalysis.

[11] Nakamoto S. (2008). Bitcoin: A peer-to-peer electronic cash system. Decentralized Bus. Rev..

[12] Yaga D., Mell P., Roby N., Scarfone K. (2019). Blockchain technology overview. arXiv.

[13] Shrimali B., Patel H.B. (2022). Blockchain state-of-the-art: Architecture, use cases, consensus, challenges and opportunities. J. King Saud Univ. Comput. Inf. Sci..

[14] Bodemer O. (2023). Blockchain for IoT: A Critical Review of the State-of-the-Art, Challenges, and Future Prospects. Authorea Prepr..

[15] Almalki J. (2023). State-of-the-Art Research in Blockchain of Things for HealthCare. Arab. J. Sci. Eng..

[16] Dai H.N., Zheng Z., Zhang Y. (2019). Blockchain for Internet of Things: A survey. IEEE Internet Things J..

[17] Khan A.A., Laghari A.A., Shaikh Z.A., Dacko-Pikiewicz Z., Kot S. (2022). Internet of Things (IoT) security with blockchain technology: A state-of-the-art review. IEEE Access.

[18] Zhao W., Yang S., Luo X. (2025). Blockchain-Facilitated Cybersecurity for Ubiquitous Internet of Things with Space–Air–Ground Integrated Networks: A Survey. Sensors.

[19] Yang Q., Wang H., Wang T., Zhang S., Wu X., Wang H. (2021). Blockchain-based decentralized energy management platform for residential distributed energy resources in a virtual power plant. Appl. Energy.

[20] Rajasekaran A.S., Azees M., Al-Turjman F. (2022). A comprehensive survey on blockchain technology. Sustain. Energy Technol. Assess..

[21] Taherdoost H. (2023). Smart Contracts in Blockchain Technology: A Critical Review. Information.

[22] Christidis K., Devetsikiotis M. (2016). Blockchains and smart contracts for the internet of things. IEEE Access.

[23] Raikwar M., Gligoroski D., Kralevska K. (2019). SoK of used cryptography in blockchain. IEEE Access.

[24] Mohammed A., Salah K., Jayaraman R., Simsekler M., Shehata A., Al-Fuqaha A. (2021). Blockchain-Based Supply Chain Traceability: Tokenization, Digital Twins, and Oracles. IEEE Access.

[25] Wood G., Fisher R., Wilke P. (2025). Polkadot: A Hybrid Consensus Protocol for Scalable and Secure Multi-Chain Networks. J. Distrib. Ledger Technol..

[26] Zhao L., Chen Y., Wang B. (2023). Hybrid Consensus Mechanisms for Blockchain Security and Scalability: A Comparative Study. Nat. Commun. Comput..

[27] Machado A.T., Yamaguti R., Assumpção R.M., Branquinho O.C., Cardieri P. Application of Three-Phase Methodology for Retrofit 4.0 in Legacy Industrial Plants. Proceedings of the IARIA Congress 2023: The 2023 IARIA Annual Congress on Frontiers in Science, Technology, Services, and Applications.

[28] Li Q., Chen Y.L. (2009). Entity-relationship diagram. Modeling and Analysis of Enterprise and Information Systems.

[29] Sood R., Kaur H. (2023). A literature review on rsa, des and aes encryption algorithms. Emerging Trends in Engineering and Management.

